# IAA producing fungal endophyte *Penicillium roqueforti* Thom., enhances stress tolerance and nutrients uptake in wheat plants grown on heavy metal contaminated soils

**DOI:** 10.1371/journal.pone.0208150

**Published:** 2018-11-29

**Authors:** Muhammad Ikram, Niaz Ali, Gul Jan, Farzana Gul Jan, Inayat Ur Rahman, Amjad Iqbal, Muhammad Hamayun

**Affiliations:** 1 Department of Botany, Hazara University, Mansehra, Khyber Pakhtunkhwa, Pakistan; 2 Department of Botany, Abdul Wali Khan University Mardan, Khyber Pakhtunkhwa, Pakistan; 3 Department of Agriculture, Abdul Wali Khan University Mardan, Khyber Pakhtunkhwa, Pakistan; Universita degli Studi del Piemonte Orientale Amedeo Avogadro, ITALY

## Abstract

Heavy metals contaminated soil is a serious environmental concern that has a negative impact on agriculture and ecosystem. Economical and efficient ways are needed to address this problem worldwide. In this regard, exploration and application of proficient microbial strains that can help the crop plants to thrive in agricultural soils that are greatly contaminated with heavy metals. The present study mainly focused on the effect of IAA producing endophytic fungi *Penicillium ruqueforti* Thom., on wheat plants cultivated in soil rich in heavy metals (Ni, Cd, Cu, Zn, and Pb). *P*. *ruqueforti* has induced great resistance in wheat inoculated plants grown in heavy metal contaminated soil. Application of the isolated strain of *P*. *ruqueforti* restricted the transfer of heavy metals from soil to the plants by secreting indole acetic acid (IAA). Furthermore, *P*. *ruqueforti* inoculated wheat seedlings watered with waste water had higher plant growth, nutrient uptake and low concentrations of heavy metals in shoot and roots. On the contrary, non-inoculated wheat plants under heavy metal stress had stunted growth with symptoms of chlorosis. From the results, it is concluded that *P*. *ruqueforti* inoculation can establish a symbiotic relationship with host plants, which is useful for phytostabilization of heavy metals or in other words helping the host crops to flourish through soil that are highly contaminated with heavy metals.

## Introduction

Heavy metal contamination is among one the global challenge and their enhanced concentration poses a significant risks to plants, ecosystem and public health [1, 2]. In addition, access of heavy metals in soils result in decreased soil fertility that in turn effects plant growth, yield and vigor [3]. Therefore, the development of novel environmental conservation and soil remediation strategies can serve as a paramount in securing healthy environment [4]. Though, small amounts of heavy metals are obligatory for cellular activities, but its presence in excessive amounts in the environment can be associated with mining, improper handling of industrial wastes, agricultural fertilizers and pesticides [5, 6].

The heavy metals persist in the soils of industrial areas or other parts with mining facilities and that is why, biological and natural reclamation of such polluted sites are challenging [7–10]. In order to clean the soil of such areas, the heavy metal may be extracted through electrochemical methods, thermal processes, washing, stabilization/solidification and burial tie, etc. Though, the methods are sophisticated, but they are quiet expensive to reclaim the soil for crop production purposes [11]. Keeping feeding of growing population of the world in view, an alternative strategy is demanded to make the heavy metal rich soil fit for crop production. In the current scenario, the use of plant beneficial microorganisms (especially endophytes) that interacts with plants in rhizoshpere and enabling them to alter the uptake, mobility and bioavailabity of metals ion seems the best [12–16]. These microbes release plant growth promoting substances such as siderophores, phosphate solubilzation, growth hormones IAA, and 1-aminocyclopropane-1-carboxylic acid (ACC) deaminase that improves growth of plants in metal contaminated soils [17–22]. Furthermore, microbial activities inside host roots increases efficiency of metal degradation in contaminated soil through phytostabalization (by reducing the mobility of heavy metals in to the host plants), enhances metal tolerance and biomass production in host plants [23].

Endophytic microbes (fungi and bacteria) involved in phytoremediation process have shown tremendous potential in improving phytoremediation processes by reducing metal availability, altering soil pH, releasing chelating agents along with multiple enzymatic properties [24–26]. More recently, various studies have described the role of beneficial endophytic fungi in abiotic stresses [27, 28], however the field is still wide open to discover potential endophytic strains with a role in reducing heavy metal stress in crop plants. Therefore, the objective of the present work was: To isolate and identify beneficial endophytic fungi from *Solanum surattense* Burm. f. that can help the host plants under heavy metals stress; To assess the strain(s) for its role in ameliorating the effect of metal ions and enabling the host plants to take up the available nutrients. We reckon that this is the first ever report on isolation of endophytic fungi from the roots of *S*. *surattense* and confirming its potential in phytoremediation and stress adaptation.

## Materials and methods

### Climatic condition of the study area

Gadoon amazai is located in the Northwest of Pakistan, with latitude 34.120155" north and longitude 72.470154" east with an elevation of about 341 meters above the sea level. Rainfall occurs in both winter and summer varies from 456 to 639 mm due to monsoon, which comes from Malakand and Hazara regions. Average temperature ranges from 2 °C to 40 °C with semi-arid climate, i.e. hot summers and cold winters. Winter starts in mid-November and ends in March, while summer starts from May and ends in September. Such variation produce diverse microbial habitat to adapt various environmental conditions and nature has bestowed the region to study best source of ecologically varied microorganisms.

### Plant material, soil, water sampling and chemical analysis

The current study was approved by the “Advanced Studies and Research Board (AS&RB), Hazara University Mansehra, Pakistan” and this study did not involve any endangered or protected species. Healthy plants of *S*. *surattense* Burm. f. were collected from the industrial sites of Gadun, Khyber Pakhtunkhwa, Pakistan (S1 Fig). Seeds of wheat variety Bhakkar-2000 (KJ672075) were obtained from the Department of Crop Science, National Agriculture Research Centre (NARC), Islamabad, Pakistan. Similarly, seeds of Auxin-deficient mutant kernal (*dek 18*) and wild kernal (*Dek 18*) maize developed by University of Hohenheim, Germany were employed as a control in IAA assays. Soil and water samples were collected from the same area in sterile plastic bags and taken to the laboratory for analysis. To determine heavy metals, water samples were mixed and kept overnight with 10% hydrochloric acid solution. Few drops of HNO_3_ were also added in each sample and stored at 5 °C until analyzed. Soil samples were collected from a depth of 0-12cm with sterile stainless steel auger, the soil samples were air dried and kept covered to avoid contamination. The dried soil samples were grinded and passed through 200 mech sieve [29]. These soil samples were then autoclaved and filled into 1kg pots. Plant samples were washed with distilled water to remove any dirt, oven dried for 48 h and finally grounded. Subsequent sample preparations and analysis were done as described by Turkdogan et al., [29].

### Isolation, surface sterilization and screening of endophytic fungal strains

The fresh samples were processed in the laboratory within 48 h of collection. Surface sterilization was performed by the method of Qin et al., [30]. The root segments of collected *S*. *surattense* were washed (tap water) to remove soil particles, than soaked for 5 min in distilled water having few drops of tween 80. After washing, the root samples were cut into small segments (0.5 cm), washed twice with autoclaved distilled water (ddH_2_O). The segments were surface sterilized with 70% ethanol and further treated with 1% perchloric acid, (30 s), rinsed with autoclaved distilled water and dried under sterile condition. The outer layer of the sterilized root segments were then removed and 5 segments per plate was placed on Hagem medium (0.05% KH_2_PO_4_, 0.05% MgSO_4_·7H_2_O, 0.05% NH4Cl, 0.5% glucose, 80 ppm streptomycin, 0.1% FeCl_3_ and 1.5% agar; pH 5.6 ± 0.2) in order to isolate endophytic fungi [31]. The plates were sealed and incubated at 28 °C for one week to observe the growth of endophytic fungi. Intact surface sterilized roots were also checked for any contamination according to the method described by Schulz et al., [32], which confirmed the absence of any microbial growth on media plates. After incubation, the endophytic fungal hyphae that grew out from the root tissues were collected by Pasteur pipettes and transferred to a new PDA (potato dextrose agar) plates. The plates having the harvested hyphae were incubated for another 7 days at 25 °C to get pure cultures. Czapek culture broth (1% peptone, 1% glucose, 0.05% MgSO_4_·7H_2_O, 0.05% KCl, 0.001% FeSO_4_·7H_2_O; pH 7.3 ± 0.2) was used for further purification of fungal isolates, shaking at 120 rpm for 7 days at 30 °C. The culture filtrate (CF) was harvested through centrifugation at 4,000 ×g at 4 °C for 15 min. Harvested pellet and supernatant were lyophilized (ISE Bondiro Freeze Dryer) and kept at -70 °C.

The pellet was used for identification of fungal isolate, while the supernatant was diluted in 1 ml of ddH_2_O to perform the screening experiment. To assess the plant growth promoting/inhibiting capacity of the fungal isolates, seeds of IAA mutant kernel (*dek18)* and wild type kernel (*Dek18)* maize line were surface-sterilized with 2.5% sodium hypochlorite for 30 min, rinsed with ddH_2_O, and then incubated for 48 h at 28 °C. Germinated maize seedlings were transplanted to autoclaved pots containing 0.8% water-agar medium and kept in a growth chamber for further growth. After attaining the two leaves stage, 10 μl of supernatant solution of fungal CF was applied at the seedling apex. After one week of treatment, the shoot length, chlorophyll content, and shoot fresh and dry weights were recorded and compared with negative (treated with DDW).

### Screening for auxin production

The production of IAA was calorimetrically measured for all isolated strains as previously described [33], using Salkowski’s reagent [34]. To 1 mL of centrifuged broth culture supernatant, 2 mL of Salkowski reagent was added and the mixture was incubated in the dark for 30 minutes. Absorbance of the pink colored complex was taken at 540 nm against the standard IAA using UV/VIZ spectrophotometer.

### IAA determination by HPLC

IAA was further quantified through HPLC following the method of Khan et al., [35] with minor modification, *i*.*e*. injecting 20 μl fungal extract into a 5 μm reverse phase column (Waters Associates Bondapak C18, 250 mm × 4 mm) in a Waters Associates liquid chromatograph. Samples were analyzed under isocratic conditions with MeOH and water (80:20 v/v) as separation solvent at 1,200 p.s.i. flow rate of 1.0 ml/min. Elutes were detected by differential ultraviolet detector at 254 nm. IAA was quantified by peak area reference obtained from IAA standard.

### Screening of isolated strains for NaCl and heavy metals stress

Isolated strains were tested against heavy metals resistance by the method of Cervantes et al., [36]. Soluble metals such as (CuSO_4_), cadmium (CdSO_4_), nickel (NiSO_4_), zinc (ZnSO_4_) and lead (PbSO_4)_ were added to freshly prepared agar inoculated plates at various concentrations, ranging from 50 to 350 μg/ml and kept overnight at 28_°C in dark. The growth of isolated strains were measured as by the method of Hamayun et al. [37]. The strain that were mostly active under heavy metal stress were selected and grown in broth to determine the metal uptake potential of the selected strains.

To observe NaCl tolerance, the isolated endophytic fungus (24 h old test culture) was subjected to various salt concentrations (0, 50, 100, 150, 200, 250, 300, 350, 400, 450 and 500 mM) to check its tolerance. The cultures in a salt medium were incubated at 27 °C with shaking (120 rpm) for 24–48 h and the growth was measured as described by Kumar et al., [38]. The strain that performed best were selected and grown in Czapek broth for 7 days at 27_°C in a shaking incubator with 120 rpm for further experiments.

### DNA extraction and fungal isolate identification

#### Morphological features of isolated strains

The endophytic fungal culture was morphologically identified based on their growth characteristics, included color and shape, mycelium type and conidiophore structure. Microscopically the strains spore, hyphae and reproductive structures were identified through lacto phenol staining technique by light microscope (Stemi SV 11 Apo, Carl Zeiss) by the method of Nagamani et al., [39] Macroscopic photographs were taken by digital camera (Canon Co., Ltd, Japan).

#### DNA extraction of isolated strains

Fungal mycelium (200 mg) were collected from fresh culture, suspended in 500 μl of a bead beating solution with addition of 5% sodium dodecyl sulfate mixed in 1.5 mL micro centrifuge tubes. The tubes were then vortexed and centrifuged for 10 min at 11,000 g at 4 °C. The supernatants were decanted into new tubes and equal volumes of phenol: chloroform: isoamyl alcohol (25:24:1) was added to each sample, again vortexed and centrifuged for 5 min. The aqueous layer was transferred to a new tube an equal volume of chloroform: isoamyl alcohol (24:1) were added to it and centrifuged for 5 min at 10,000 g. The supernatant was transferred to the new eppendorf tubes, and 2.5 volumes of isopropanol were added for precipitation of DNA. The tubes were incubated in a refrigerator for 1 hour, and centrifuged for 10 min at 14,000 g. The pellets were washed twice with cold 70% ethanol, air-dried and added 40 μl TE buffer. The purity of the extracted DNA and its quantity was measured by Thermo Scientific Nano Drop spectrophotometer at 260 nm [40].

#### PCR-amplification and 18S rDNA sequence analysis

DNA isolation and PCR of selected strain were performed, according to the protocol of Khan et al., [41], with some modification. Selected endophytic fungal strain was identified by amplifying their ITS region of 18 S rDNA with universal primers, NS1 5' (GTA GTC ATA TGC TTG TCT C) 3' [42] and NS24 5' (AAA CCT TGT TAC GAC TTT TA) 3' [43]. The PCR reaction was performed with 20 ng of genomic DNA as the template in a 30 μl reaction mixture by using a EF-Taq (SolGent, Korea) as follows: 95 °C for 2 min; 35 cycles (95 °C for 1 min, 55 °C for 1 min and 72 °C for 1 min); 72 °C for 10 min. The PCR products along with DNA markers (DNA ladder) were then loaded onto an agarose gel and subjected to ectrophoresis for 30 minutes. The gel was finally stained with 0.01 g/ml ethidium bromide and examined under UV trans-illuminator lamp.

#### Sequencing of isolated strains

Approximately, 1600 bp purified PCR products were sequenced with 18S rDNA region by using universal primers NS1 5' (GTA GTC ATA TGC TTG TCT C) 3' [42] and NS24 5' (AAA CCT TGT TAC GAC TTT TA) 3' [43], accomplished through Big Dye terminator cycle sequencing kit v.3.1. Both PCR sequencing and amplification was analysed by an automated DNA sequencing system (Applied Biosystems, Foster City, USA) at the Macrogen, Inc., Seoul, Korea.

### Wheat plants germination, pot inoculation and harvesting

Healthy seeds of wheat variety Bhakkar-2000 (KJ672075) were surface sterilized with 70% ethanol for 1 min followed by 1% perchloric acid for 30 sec and rinsed thrice with ddH_2_O. The seeds were then incubated to germinate for 24 h at 28 °C in petri plates. To investigate the wheat plants association with microbes under heavey metal stress, the germinated seeds from petri plates were transferd to autoclaved pots containing 3 kg soil and grown for 4 weeks in greenhouse with day light of 12 h and temperature of 22–35 °C. The experiment was carried out in triplicate, each replicate comprised of 10 pots and each pot contained 6 seedlings (total = 6 × 10 × 3 = 180 seedlings per treatment). After one week of growth, the bioactive strain (metal-resistant) grown in Czapek broth (20 ml containing 3 g of fungal mycellium) was applied to the plants according to the Khan and Lee [35]. After 15 days of growth, 80 ml of waste water collected from industrial sites of Gadun was applied to the plants for 2 weeks at 48 h of interval. The plants were finally harvested after 4 weeks for subsequent analysis. Endophyte non-inoculated wheat plants under similar conditions and setup were used as a control. To see endophytic fungal colonization potential in plant tissues, 0.5–8 mm slices of wheat roots and shoots were visualized using light microscope (Stemi SV 11 Apo, Carl Zeiss).

### Physiological analysis of growth attributes and biomolecules

Chlorophyll a, chlorophyll b, and carotenoids were analyzed by the method of Duxbury and Yentsch, [44]. Fully expanded fresh leaves (0.8-cm) of fungal associated and non-inoculated wheat plants were homogenized with 2 mL of acetone (80%), respectively and washed twice to reach final volume of 7 ml. The absorbance was measured using a spectrophotometer at 480 nm, 645 nm, and 663 nm.

Total sugar was measured by the method of Dubois et al., [45]. Fresh leaf samples (0.2 g) were homogenized in 4 ml of water and boiled in a water bath at 100 °C for 30 min. The contents were then centrifuged at 12,000 rpm for 10 min. The supernatant was diluted with water to 10 mL and 0.2 ml of anthrone solution (0.5 g anthrone dissolved in 500mL of 80% sulfuric acid solution) was added. The mixture was incubated at 100 °C for 10 min and finally the absorbance was measured at 620 nm.

Malondialdehyde (MDA) contents were determined by the procedure of Lowry et al., [46]. Fresh leaf samples (0.2 g) were homogenized in 10 ml of 10% trichloroacetic acid and centrifuged at 12000 rpm for 10 min. The supernatant (2 ml) was added to 2 ml of 0.6% thiobarbituric acid (TBA) and incubated in a water bath for 15 min at 100 °C. The mixture was cooled and centrifuged at 12,000 rpm for 10 min. The supernatant was measured at 532, 600, and 450 nm.

Proline contents was measured by the method of Bates et al., [47] with minor changes. Fresh leaf samples (0.2 g) were homogenized in 5 ml of 3% aqueous sulphosalicylic acid and then centrifuged at 12,000 rpm for 10 min. Acid ninhydrin (2 ml) and glacial acetic acid (2 ml) were added to the 2 ml of supernatant. The mixture was boiled in a water bath at 100 °C for 1 h and then extracted with 4 ml of toluene. The absorbance of chromophore was measured at 520 nm using toluene as a blank. Chlorophyll content was measured with SPAD analyzer while, net transpiration rate, stomatal conductance, shoot fresh and dry weights, leaf length, breadth and area were analyzed with Vernier caliper (ECV150C) following Mateos-Naranjo et al. [48].

### Antioxidant enzymes activity in plants

Enzymatic activities including catalase (CAT), ascorbate (APX), peroxidase POD and reduced glutathione (GSH) were analyzed using spectrophotometer. Wheat plants were harvested after 30 days of sowing and stored at -70 °C for enzyme activities.

Activity of POD was determined by the method of Gorin and Heidema, [49]. The assay comprised of a mixture containing phosphate buffer 125 mM, pH 6.8, pyrogallol 50 mM, H_2_O_2_ 50 mM and 1 ml diluted enzyme extract. The mixture was incubated at 25 °C for 5 min and the reaction was stopped by adding 0.5 ml of 5% H_2_SO_4_. The amount of purpurogallin formed was observed at 420 nm.

APX activity was determined by monitoring ascorbate oxidation at 290 nm. The reaction mixture consisted of 35 μl enzyme extract, 0.965 ml of 50 mM potassium phosphate buffer: pH 7.0, 1mM H_2_O_2_, 0.8 mM L-ascorbic acid [50]. The mixture was incubated for 1 min and the absorbance was measured at 290 nm.

The CAT activity was determined according to the method of Goel and Sheoran, [51]. The reaction mixture comprised of 20 μl enzyme extract and 0.980 mL of 50 mM potassium phosphate buffer, pH 7.0, with 20 mM H_2_O_2_ and was incubated at 28 °C for 1 min and the absorbance was computed at 240 nm.

GSH activity was determined by measuring the oxidation of NADPH at 340 nm in a reaction mixture containing 1.8 ml phosphate buffer, 300 μl each of EDTA, NADPH, oxidized glutathione (GSSG) and enzyme extract. The decrease in absorbance per minute was followed at 340 nm [52].

### Metal contents in plants

At the end of the experiment, shoots and root of fungal inoculated and non-inoculated wheat plants were analyzed for macro and micronutrient contents. Plant tissues were, grinded and digested in HNO_3_ and H_2_O_2_ through microwave digestion system (CEM-MDS 2000), and analyzed for macro-nutrient contents (Mg, K, Na, Ca) and metals contents including (Ni, Cd Cu, Zn, and Pb) by using Atomic Absorption spectrometer (SSD/DN/119/01). Plant tissues were also analyzed for total phosphorus contents as described by Ryan et al., [53].

### Measurement of plant transfer factor and pollution load index (PLI)

Metal concentrations of soils and plants extracts were measured, and plant concentration factor (PCF) was calculated on dry weight basis as per Cui et al., [54].

$$PCF=C_{plant}/C_{soil}$$

where Cplant and Csoil represent the heavy metal concentration in extracts of plants and soils on dry weight basis, respectively.

Similarly, the amount of soil contamination/pollution or pollution load index (PLI) was measured through the equation of Liu et al. [55] for specific metals concentration.

$$PLI=\frac{C_{soil}(Sample)}{C_{reference}(Reference)}$$

where Csoil (Samples) and Creference (Reference) represent the heavy metal concentrations in the wastewater-irrigated and reference soils, respectively.

### Quality control analysis

Quality control analysis was also carried out in triplicates (n = 3), by repeated measurements of each sample. For verification and accuracy of results, standard reference soil (NIST, 2709 San Joaquin) and plant tissues (NIST, 1547 Peach leave) of National Institute of Science and Technology were included in each sample batch. Blank samples were run in the digestion procedure.

### Computational analysis

For DNA based data, multiple sequence alignment was performed using Clustal W alignment tool, and phylogenetic analysis was carried out using Neighbour joining (NJ) method with bioinformatics software Geneious R6 [56]. Nodal support was assessed via bootstrapping, and the bootstrap consensus tree was inferred from 1000 replicates [57]. The isolated sequence (18S ribosomal DNA) of the entophytic fungal strain was submitted to NCBI GenBank under Accession number KY100257.

### Statistical analysis

All the experiments were replicated (each replicate consisted of 5-seedlings) three times and the data that is presented as mean was followed by ±SD. The significant differences (P < 0.05) among the paired means were computed by ttest using Microsoft Excel, 2010, whereas the significant differences (P < 0.05) among the various means were computed by Tukey HSD test.

## Results

### Isolation, screening and identification of potential fungal strain from halophytic plant

**Fig 1:**
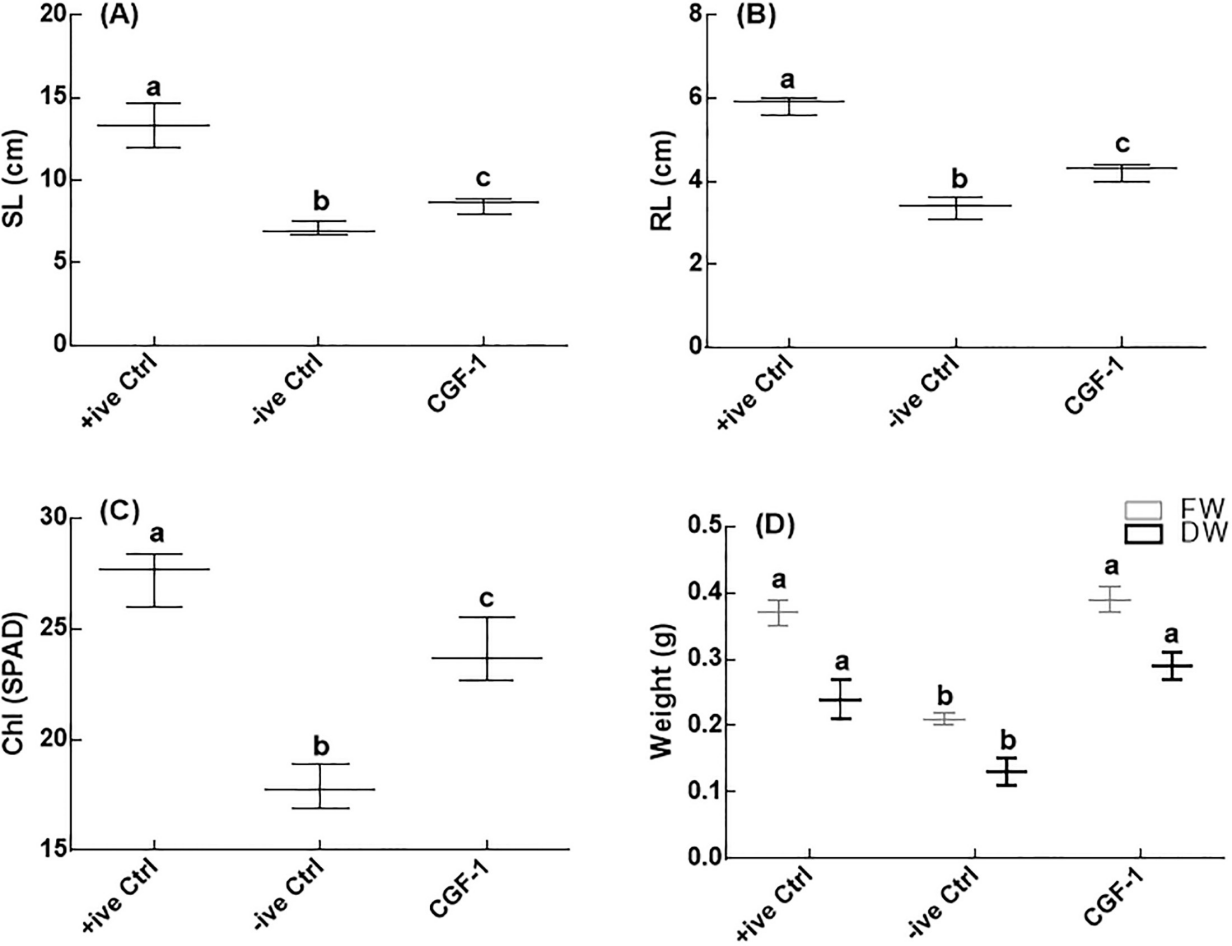
Plant growth promoting activity of isolated fungal strain CGF-1 from halophytic plant *S*. *surattense* Burm.f. +ive control = IAA mutant kernal (*dek 18*) maize line treated with distil water; -ive control = wild *kernal* (*Dek 18*) maize line grown under metal stress; CGF1 was applied to wild *kernal* (*Dek 18*) maize line grown under metal stress. Fig 1A represents shoot length; Fig 1B represents root length; Fig 1C represents chlorophyll content; Fig 1D represents weight of the plants; SL = shoot length; RL = root length; Chl = chlorophyll. The experiment was carried out in triplicate, each replicate comprised of 10 pots and each pot contained 6 seedlings (total = 6 × 10 × 3 = 180 seedlings per treatment). The boxplots having different letters are significantly different from each other at P < 0.05.

**Fig 2:**
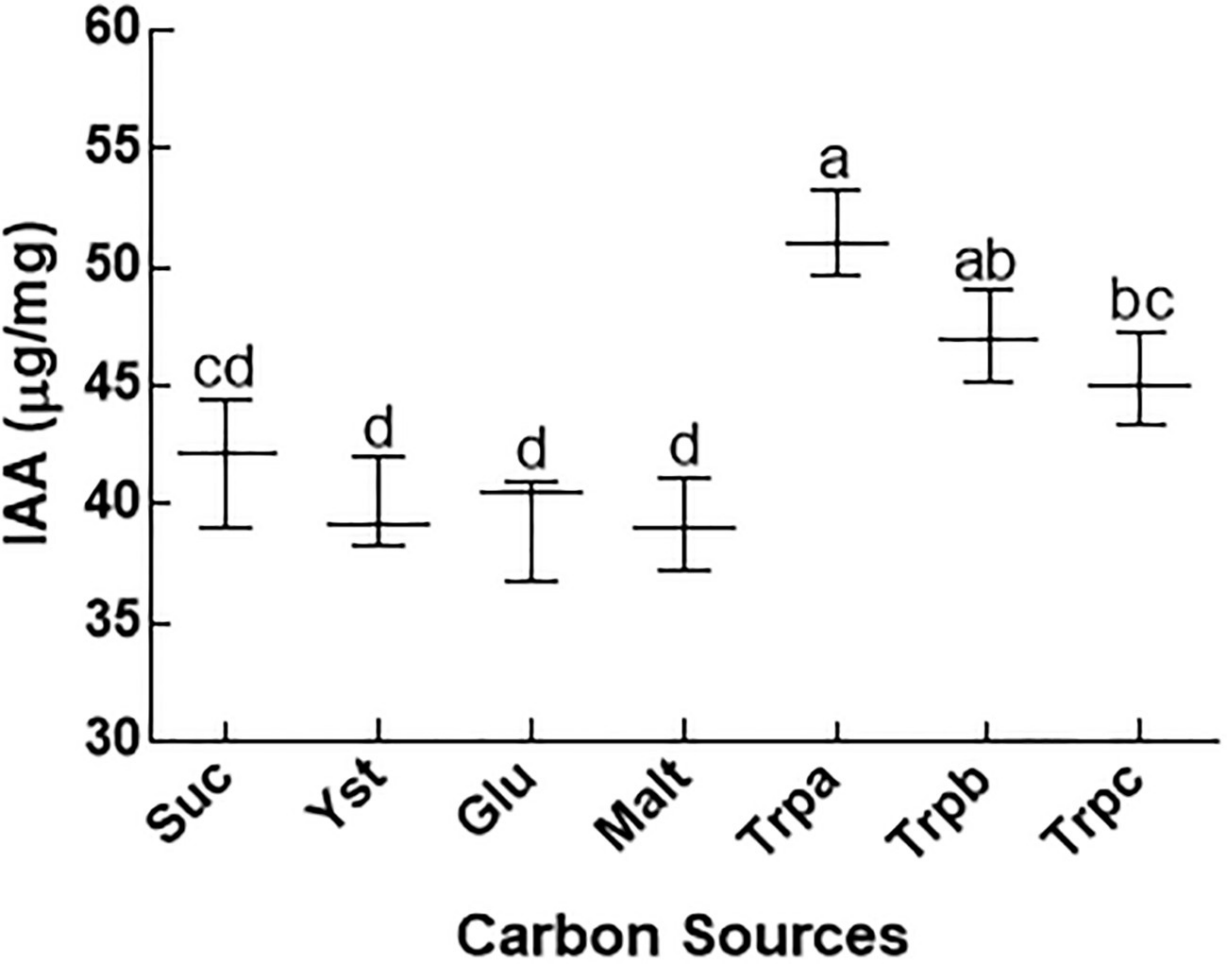
IAA estimation (μg/ml) in endophytic fungal strain CGF-1. CGF-1 strain was grown on different sources and the produced IAA was quantified by using HPLC. Suc = sucrose; Yst = yeast; Glu = glucose; Malt = maltose; Trpa = 1000 μg tryptophan; Trpb = 500 μg tryptophan; Trpc = 100 μg tryptophan. The experiment was carried out in triplicate. The boxplots having different letters are significantly different from each other at P < 0.05.

CGF1 an endophytic fungal strain was isolated from the root pieces of halophytic plant *S*. *surattense* Burm. f. after cultured for seven days on Hagem media. The separated fungal stain was identified on the basis of height and aerial hyphae colour, shape, surface texture and margin characteristics. IAA mutant kernel (*dek18)* and wild type kernel (*Dek18)* of maize were used to see the potential of growth promoting/inhibiting IAA secreting strain. Wild maize plants (*Dek18)* inoculated with CGF-1 strain had the highest shoot, and root length, chlorophyll contents, fresh and dry weight of shoots as compared to negative control (Fig 1). The growth promoting response of CGF-1 suggested the presence of IAA (a growth enhancing substances) that ameliorated the seedlings growth of kernel (*Dek18*). The CGF-1 strain was then grown on various extracts to see its effect on IAA production capapbility of the CGF-1 strain. The HPLC analysis revealed that the amount of IAA was higher in Trpa extract (51.3 μg/ml), followed by Trpb (47.1 μg/ml) and Trpc (45.1 μg/ml). The lowest concentration (38.7 μg/ml) of IAA by CGF-1 was recorded in sucrose extract (Fig 2).

### Resistance of isolated strains toward salt and heavy metals

**Fig 3:**
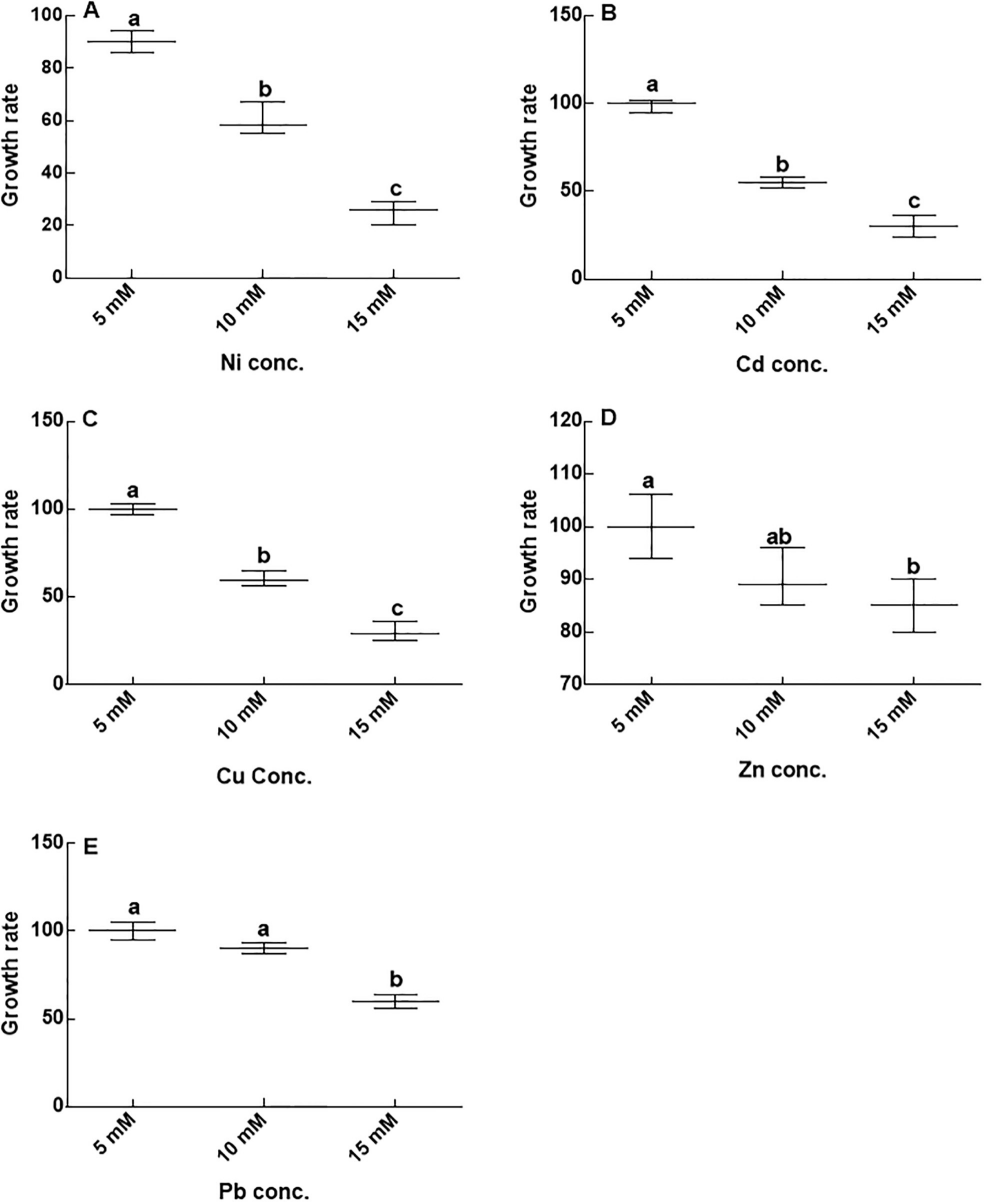
Metal resistance assay of CGF-1. Fig 3A = growth of CGF-1 on various concentration of nickle; Fig 3B = growth of CGF-1 on various concentration of cadmium; Fig 3C = growth of CGF-1 on various concentration of copper; Fig 3D = growth of CGF-1 on various concentration of Zn; Fig 3E = growth of CGF-1 on various concentration of lead; Ni = nickel; Cd = cadmium; Cu = copper; Zn = zinc; Pb = lead. The experiment was carried out in triplicate. The boxplots having different letters are significantly different from each other at P < 0.05.

**Fig 4:**
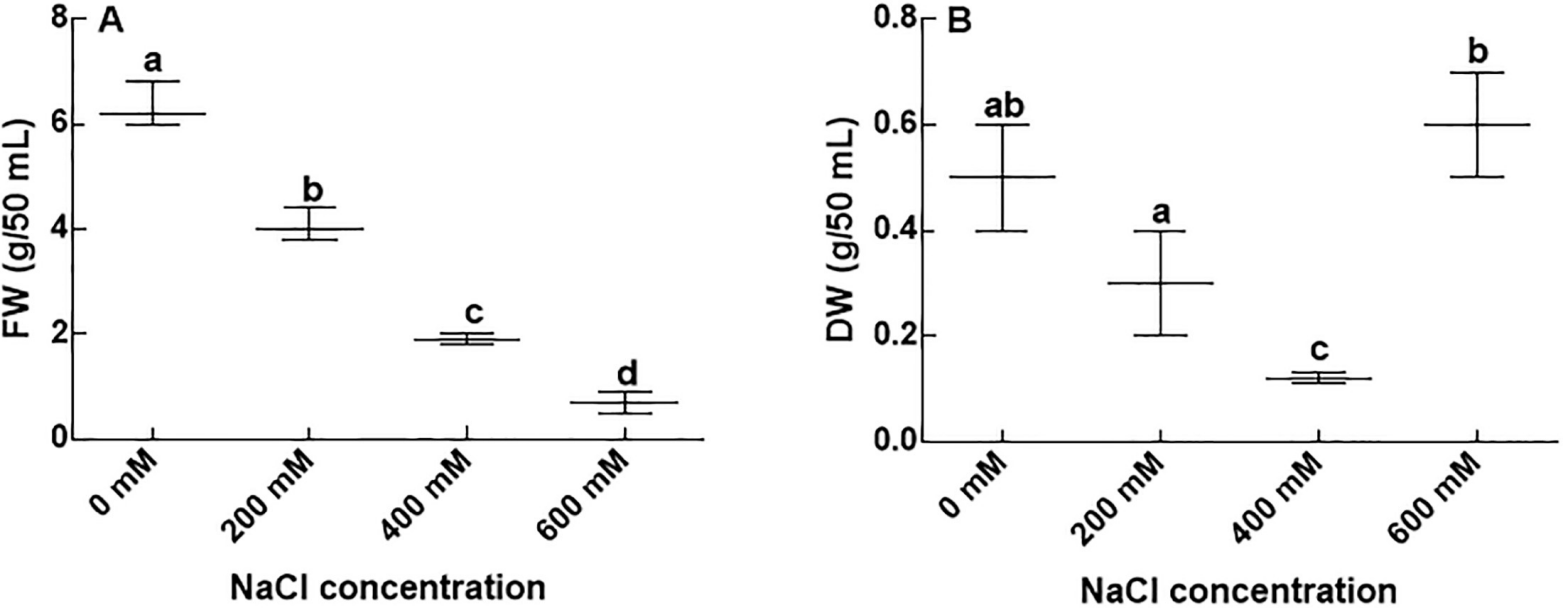
NaCl resistance assay of CGF-1. Fig 4A = fresh weight of CGF-1 grown on various concentration of NaCl; Fig 4B = dry weight of CGF-1 grown on various concentration of NaCl; FW = fresh weight; DW = dry weight. The experiment was carried out in triplicate. The boxplots having different letters are significantly different from each other at P < 0.05.

Resistance of the isolated CGF-1 strain to several heavy metals (Ni, Cd Cu, Zn, and Pb) and various levels of NaCl has been tested. The CGF-1 tolerated the various concentration (5 mM, 10 mM and 15 mM) of heavy metals that has been supplemented in agar medium (Fig 3A–3E). The CGF-1 showed highest resistance against Zn and Pb (i.e. even at 10 mM concentration) (Fig 3D and 3E), followed by Cu and Cd (Fig 3B and 3C). In contrast, Ni, resistance of the isolates was only up to 5mM (3A). Moreover, salt tolerance response of the CGF-1 assessed at 0 mM, 200 mM, 400 mM and 600 mM presented that the strain has tolerated the NaCl up to the maximum concentration (Fig 4A and 4B), which suggests that CGF-1 is halotolerant. However, at higher concentrations of salt stress the biomass of the fungal strain was significantly reduced compared to the lower concentrations.

### Identification of the fungal strain CGF-1

Two cultural filtrates of the endophytic fungal strain CGF-1 were amplified through PCR and sequenced commercially at Korean Biotechnology Laboratory, Seoul South Korea, using both NS1 5'and NS24 5' primers. To identify the CGF-1 strain, the sequenced ITS region was compared to sequences in the NCBI database by BLAST search analysis (http://www.ncbi.nlm.nih.gov/). The results revealed that the CGF-1 fungal endophyte exhibited higher levels of ITS sequence identity (99%) to *P*. *roqueforti*. Two methods were employed to construct phylogenetic tree, including maximum likelihood (ML) and neighbor-joining (NJ). The software used was MEGA 6 after ITS sequence alignment with the MAFFT (version 7.222) [58]. The results revealed that CGF-1 maximum homology with *Penicillium roqueforti* strain ATCC 10110 that was supported by relatively strong bootstrap value of 99 and 100% for NJ and ML, respectively (S2 Fig). On the basis of morphology and phylogenetic relationship, strain CGF-1 was classified as a member of the genus *Penicillium* that showed similarity to *Penicillium roqueforti* strain ATCC 10110.

### Heavy metals contents in water and soil sample collected from Gadun area

Heavy metals contents in soil, wastewater samples and endophyte inoculated-wheat and non-inoculated wheat plants irrigated with waste water are presented in Table 1. Higher amounts of Pb (0.88 mgl^-1^) and Ni (0.92 mgl^-1^) were found in wastewater samples, followed by Cd (0.81 mgl^-1^) and Cu (0.79 mgl^-1^). However in soil sample Pb (65.3 mgkg^-1^) and Zn (72.3 mgkg^-1^) content were higher as compared to Cd, Ni and Cu.

**Table 1:** Heavy metals concentration in contaminated soil as well as reference soil (mgkg^-1^) and polluted water samples (mgL^-1^).

HM	RS	HMCS	WW
**Ni**	2.30 ± 0.12	10.30 ± 0.11	0.75 ± 0.16
**Cd**	0.01 ± 0.00	2.40 ± 0.07	0.81 ± 0.01
**Cu**	8.50 ± 0.12	63.20 ± 0.14	0.79 ± 0.02
**Zn**	72 ± 1.15	92.42 ± 0.15	0.61 ± 0.01
**Pb**	2.80 ± 0.12	25.30 ± 0.11	0.88 ± 0.01

HM = heavy metal; RS = reference soil; HMCS = heavy metal contaminated soil; WW = waste water; Ni = nickel; Cd = cadmium; Cu = copper; Zn = zinc; Pb = lead.

### Effect of CGF-1 on growth parameters of wheat plant

Wheat inoculated with endophytic fungal strain CGF-1, hereafter as *P*. *roqueforti* showed enhancement in growth of wheat plants, during the heavy metal stress period of 30 days (Table 2). The inoculated plants under stress had experienced positive growth promoting effect in comparison to the non-inoculated plants. Likewise, the CGF-1 treated plants had significantly (P < 0.05) higher chlorophyll contents, root and shoots weight, shoot length and breadth. Furthermore net transpiration rate and stomatal conductance were also higher in inoculated wheat plants after 30 days of stress treatment.

**Table 2:** Growth parameters of fungal inoculated and non-inoculated wheat plants under heavy metal stress.

Treatment	FIWP	NIWP
**TR (mmol.m^-2^.s^-1^)**	4 ± 0.3ns	3.8 ± 0.4ns
**SC (mmol.m^-2^.s^-1^)**	0.18 ± 0.02*	0.12 ± 0.02
**RFW (g)**	0.96 ± 0.04**	0.73 ± 0.003
**SFW (g)**	0.89 ± 0.01***	0.61 ± 0.02
**SB (cm)**	2.10 ± 0.1*	1.80 ± 0.1
**SL (cm)**	25.30 ± 0.3***	21.40 ± 0.4
**Chl. (SPAD)**	32.1 ± 0.3***	2.62 ± 0.2

FIWP = Fungal inoculated wheat plants; NIWP = Non-inoculated wheat plants; TR = transpiration rate; SC = stomatal conductance; RFW = root fresh weight; SFW = stem fresh weight; SB stem breadth; SL = stem length; Chl. = chlrophyll. Each data point represents mean of triplicated data followed by ±SD. The means were compared by using ttest;

‘***’ represents significant difference at P = 0.0005;

‘**’ represents significant difference at P = 0.005;

‘*’ represents significant difference at P = 0.05; ns = non-significant. The experiment was carried out in triplicate, each replicate comprised of 10 pots and each pot contained 6 seedlings (total = 6 × 10 × 3 = 180 seedlings per treatment).

### Biochemical contents of wheat plant tissues

**Fig 5:**
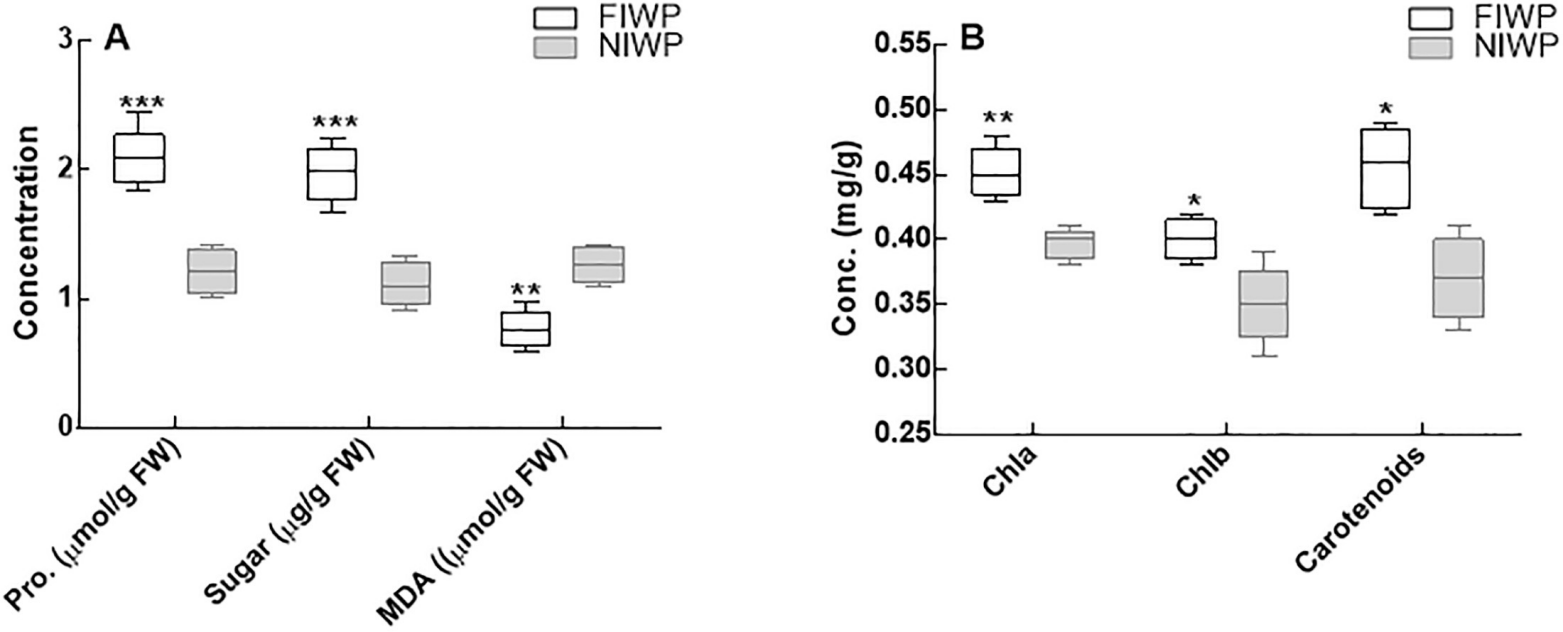
Biochemical contents of CGF-1 inoculated and non-inoculated wheat plant. Fig 5A = proline, sugar and malondialdehyde contents of CGF-1 inoculated and non-inoculated wheat plant grown under heavy metal stress; Fig 5B = Chla, Chlb and carotenoid contents of CGF-1 inoculated and non-inoculated wheat plant grown under heavy metal stress; FIWP = fungal inoculated wheat plant; NIWP = non-inoculated wheat plant; Pro = proline; MDA = malondialdehyde; Chla = chlorophyll a; Chlb = chlorophyll b. The experiment was carried out in triplicate, each replicate comprised of 10 pots and each pot contained 6 seedlings (total = 6 × 10 × 3 = 180 seedlings per treatment). Means of fungal inoculated wheat plants (FIWP) were compared with non-inoculated wheat plants (NIWP) by using ttest; ‘***’ represents significant difference at P = 0.0005; ‘**’ represents significant difference at P = 0.005; ‘*’ represents significant difference at P = 0.05.

Proline content in fungal inoculated and non-inoculated wheat plants under heavy metal stress indicated significantly higher amounts in inoculated wheat plant. On the contrary MDA content decreased with stress in inoculated host plants and there by increases resistance toward stress. Furthermore, sugar contents were also improved in fungal inoculated wheat plants during heavy metal stress (Fig 5A). Similarly, the photosynthetic pigments (chlorophyll, carotene) of fungal inoculated and non-inoculated wheat plants showed a similar trend (Fig 5B). Visible difference indicated the role of fungal association with wheat plants. Photosynthetic pigments (chla, Chlb and carotene contents) were significantly (P < 0.05) higher as compared to non-inoculated wheat plants.

### Activities of antioxidant enzymes in plants under stress

**Fig 6:**
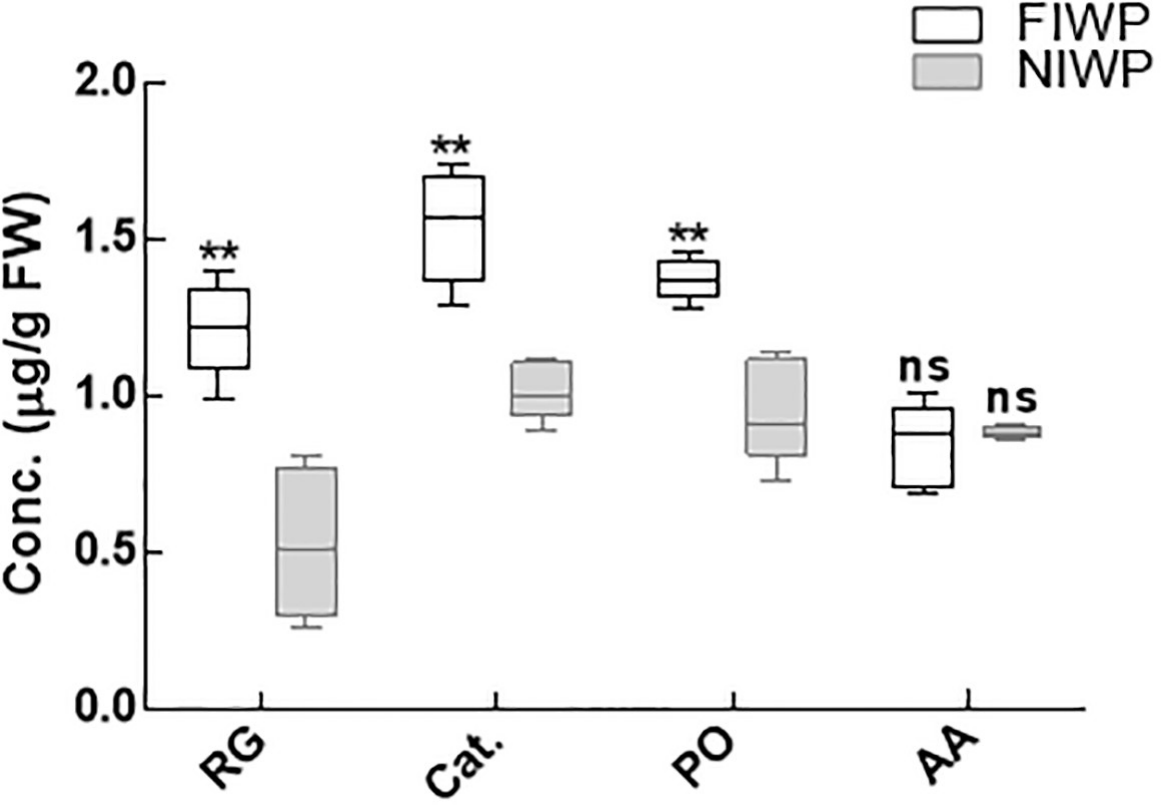
Antioxidant enzyme activities of CGF-1 inoculated and non-inoculated wheat plant. FIWP = fungal inoculated wheat plant; NIWP = non-inoculated wheat plant; RG = reduced glutathione; Cat = catalase; PO = peroxidase; AA = ascorbate. The experiment was carried out in triplicate, each replicate comprised of 10 pots and each pot contained 6 seedlings (total = 6 × 10 × 3 = 180 seedlings per treatment). Means of fungal inoculated wheat plants (FIWP) were compared with non-inoculated wheat plants (NIWP) by using ttest; ‘**’ represents significant difference at P = 0.005; ‘ns’ represents non-significant difference at P = 0.05.

Under heavy metal stress wheat plants inoculated with CGF-1 had markedly higher reduced glutathione (GSH) contents, while peroxidase and ascorbate activities were significantly (P < 0.05) reduced. Application of *P*. *roqueforti* wheat plants during heavy metals stress (waste-water treatment) had also increased the level of catalase activity (Fig 6).

### Post inoculation heavy metals concentrations in wheat plant tissues

**Fig 7:**
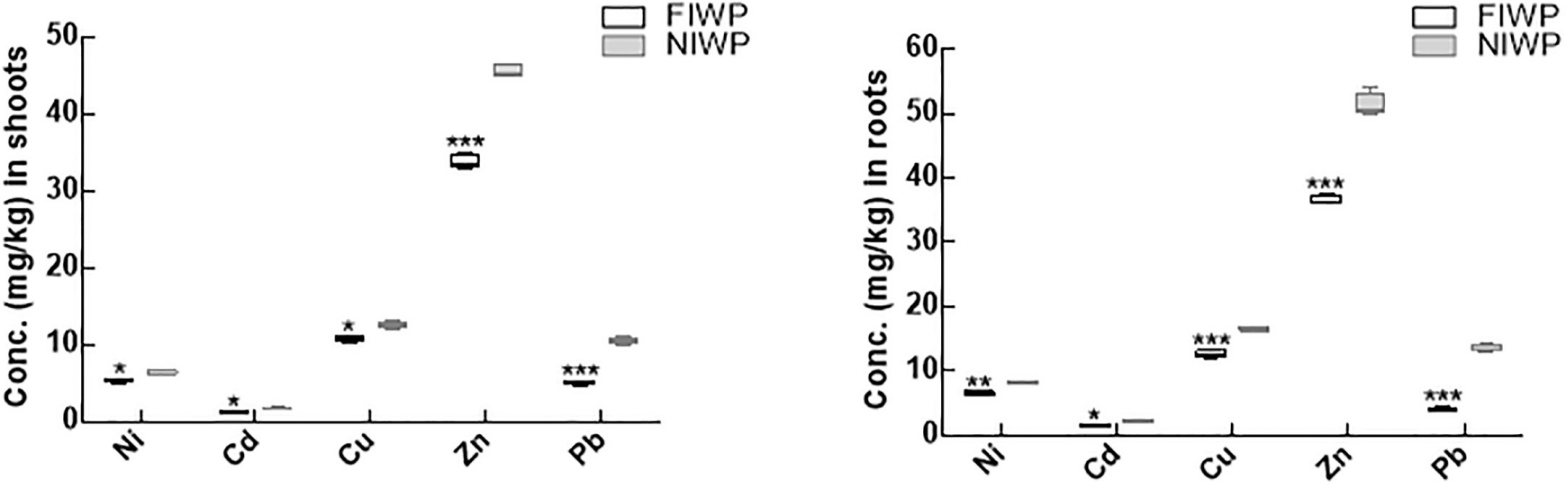
Heavy metals concentration of CGF-1 inoculated and non-inoculated wheat plants. Fig 7A represents heavy metal concentration in shoots of CGF-1 inoculated and non-inoculated wheat plant grown in contaminated soil; Fig 7B represents heavy metal concentration in roots of CGF-1 inoculated and non-inoculated wheat plant grown in contaminated soil; FIWP = fungal inoculated wheat plant; NIWP = non-inoculated wheat plant; Ni = nickel; Cd = cadmium; Cu = copper; Zn = zinc; Pb = lead. The experiment was carried out in triplicate, each replicate comprised of 10 pots and each pot contained 6 seedlings (total = 6 × 10 × 3 = 180 seedlings per treatment). Means of fungal inoculated wheat plants (FIWP) were compared with non-inoculated wheat plants (NIWP); ‘***’ represents significant difference at P = 0.0005; ‘**’ represents significant difference at P = 0.005; ‘*’ represents significant difference at P = 0.05.

After the experiment, the heavy metal concentration of both inoculated and non-inoculated wheat plants was compared (Fig 7A and 7B). The result revealed significantly (P < 0.05) higher concentration of heavy in both shoot and roots of non-inoculated wheat plants compared to CGF-1 inoculated wheat plants. The concentration of Zn and Cu was higher, followed by Pb in shoots and roots of both inoculated and non-inoculated wheat plants, (Figs 7A and 9B).

### Nutrients uptake

**Fig 8:**
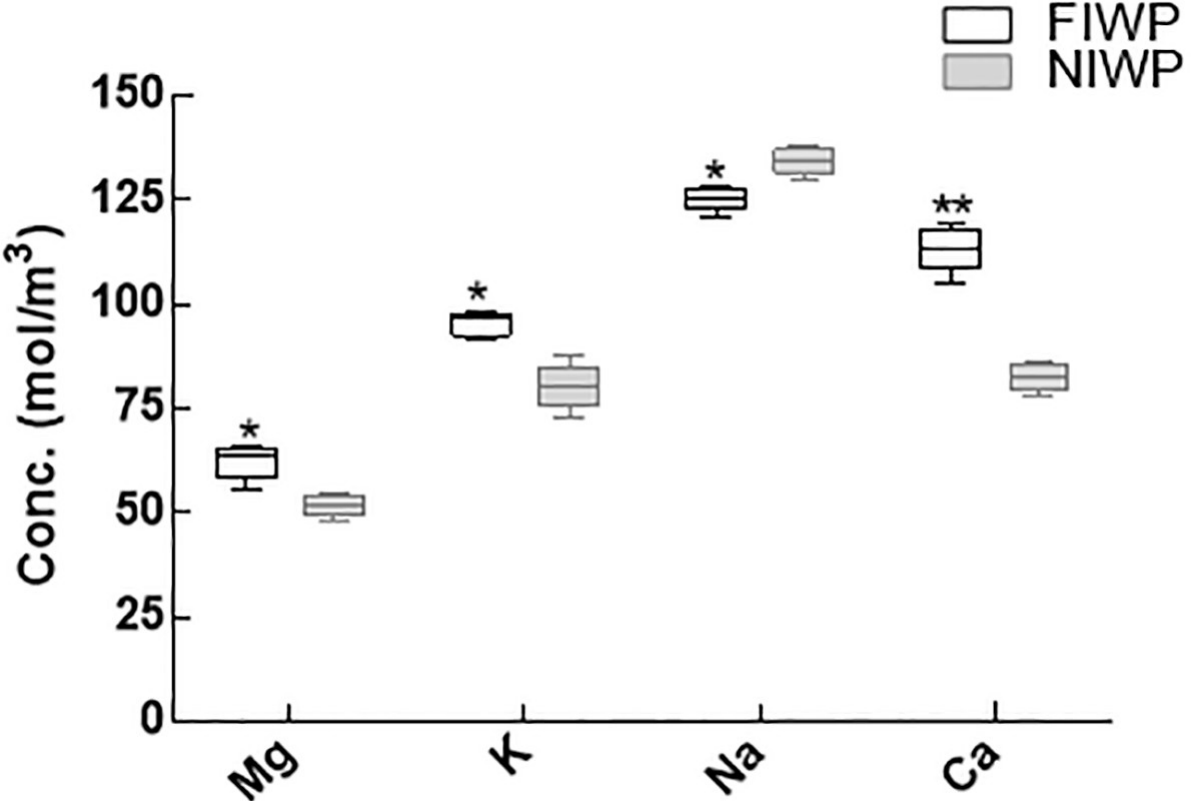
Nutrient uptake of CGF-1 inoculated and non-inoculated wheat plants. CGF-1 inoculated and non-inoculated wheat plants were analysed for the nutrient uptake from the heavy metal contaminated soil; FIWP = fungal inoculated wheat plant; NIWP = non-inoculated wheat plant; Mg = magnesium; K = potassium; Na = sodium; Ca = calcium. The experiment was carried out in triplicate, each replicate comprised of 10 pots and each pot contained 6 seedlings (total = 6 × 10 × 3 = 180 seedlings per treatment). Means of fungal inoculated wheat plants (FIWP) were compared with non-inoculated wheat plants (NIWP) by using ttest; ‘**’ represents significant difference at P = 0.005; ‘*’ represents significant difference at P = 0.05.

Concentration of nutrients (Mg, K, Na, Ca) contents of both fungal inoculated and non-inoculated wheat plants irrigated with wastewater was assessed. Overall, nutrients availability (Mg, K, Na, Ca) was high in fungal inoculated wheat plants. Excessive stress of heavy metals showed reduction in nutrients uptake by non-inoculated wheat plants. In short, endophytic association has significantly (P < 0.05) enhanced the uptake of essential nutrients (Mg, K, Na, Ca) and reduced the toxic effect of metals in host plants tissues (Fig 8).

### Pollution load index and transfer of metal from soils to wheat seedlings

**Fig 9:**
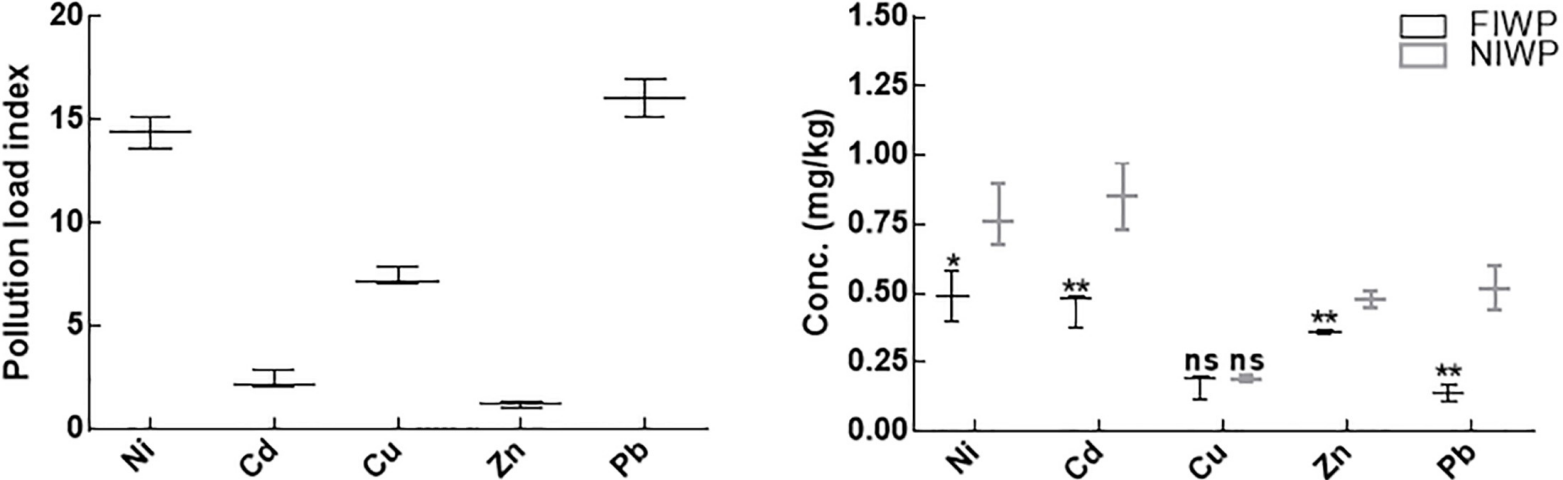
Pollution load index and pollutant concentration factor. Fig 9A represents pollution load index values of heavy metals in contaminated and reference soil; Fig 9B represents pollutant concentration factor values of heavy metals in fungal inoculated non-inoculated wheat plants in contaminated soil; FIWP = fungal inoculated wheat plant; NIWP = non-inoculated wheat plant; Ni = nickel; Cd = cadmium; Cu = copper; Zn = zinc; Pb = lead. The experiment was carried out in triplicate, each replicate comprised of 10 pots and each pot contained 6 seedlings (total = 6 × 10 × 3 = 180 seedlings per treatment). Means of fungal inoculated wheat plants (FIWP) were compared with non-inoculated wheat plants (NIWP) by using ttest; The experiment was carried out in triplicate, each replicate comprised of 10 pots and each pot contained 6 seedlings (total = 6 × 10 × 3 = 180 seedlings per treatment); ‘**’ represents significant difference at P = 0.005; ‘*’ represents significant difference at P = 0.05; ‘ns’ represents non-significant difference at P = 0.05.

PLI revealed substantial buildup of Ni, Cd Cu, Zn, and Pb, in the wastewater-irrigated soils than the reference soils. On average, the PLI indices for Ni, Cd Cu, Zn, and Pb were 4.47, 2.41, 7.4, 1.2, and 16.1, respectively (Fig 9A). Similarly, pollutants concentration factors (PCF) was compared for wastewater-irrigated fungal inoculated and non-inoculated wheat plants that exhibited significant differences at P < 0.05 (Fig 9B). Ni and Cu was highly absorbed by wheat plants irrespective of the treatment, followed by Zinc, whereas the absorption of Cu was low. The PCF mean values for heavy metals in non-inoculated wheat host plants varied significantly and absorbed in following order: Cd > Ni > Cu > Pb > Zn.

### Endophytic fungal colonization potential in wheat plant tissue

Endophytic fungal colonies were visualized in both root and shoot tissues of wheat plants using light microscope. Active association of *P*. *roqueforti* (hyphae bluish) inside host wheat plants roots and shoots were detected. The hyphae extended from epidermal cells into cortical region and reached up to the endodermis and pericycle region. Shoots were less colonized by *P*. *roqueforti* consortium as compared to roots. To double check the authenticity, endophytic fungi observed inside root and shoot tissues was cultured on PDA media and the culture was re-sequenced as described previously (S3 Fig).

## Discussion

Most plants, especially food crops are susceptible to a wide range of heavy metal stress because it can arrest plant growth, amplifies ionic imbalance inside organs and creates water deficit. Among the food crops, wheat is unequivocally recognized as a pivotal crop for addressing the growing demands of future world’s food supply. At the moment, serious efforts are underway at global level to broaden the genetic base of wheat genotypes through gene stacking and pyramided gene approaches against various biotic and abiotic stresses. Transfer of useful genes is not only time demanding, but extremely expensive and laborious. Besides, mutation within the pathogenic genes due to their rapid evolution overcomes host resistance and renders effectiveness of such introgressed useful genes [59, 60]. Therefore, exploitation of non-conventional approaches, such as the use of endophytic microorganisms for stress management and nutrients availability offers an attractive strategy. Symbiotic-interaction between endophytic fungi and higher plant has been documented in past that clearly defines the role of endophytes in plant host tolerance during stress [61, 62]. Endophytic fungi may act as a main source to secrete phytohormones and thus enhances the growth of associated plants during stress [63]. The current work was also focused on the isolation and screening of IAA producing endophytic fungi from the root of halophytic plant *S*. *surattense* and its role in stress resistance and growth-promotion of the crop plants under heavy metal stress.

Initial screening of the endophytic strain revealed a significant increase in the growth of wild maize kernel (*Dek18*) as compared to the–ive control that confirmed the presence of phytohormone, IAA. Several researches has supported the symbiotic relationship of endophytic fungi with host plant roots, where host plant can provide shelter to the endophytes and the endophytes in turn release phytoharmones to help the host [63, 64]. The association of endophytes with host plant will not only help them under normal conditions, but also under high stress conditions, including metal stress. That is the reason, endophytic fungi increases the host plant capacity to hyper accumulate heavy metals by either direct or indirect mechanisms. The endophytes can assist the host plant directly by enhanced mobilization of heavy metals, thus alleviate toxicity level of metals in plants [13, 23, 65, 66], or indirectly by improving plant growth and stress tolerance as observed in the current study. Besides plant growth promoting properties, the endophytes may benefit the host plant by rising its ability to absorb essential nutrients from the contaminated soil [67]. Furthermore, endophytes may degrad the pollutants present in the contaminated soil [68] and convert them in to a non-toxic form. Here the isolated fungal strain CGF-1 has proved to help the wheat plant under heavy metal stress and alleviate toxicity. Moreover, auxins produced by endophytes can be a contributing factor in host plant development, especially under stress conditions [69]. The exogenous supply of the phytohormones by endophytes can bring about positive physiological changes in host plant to stand the stress conditions. In addition to phytohormones, biofertilization ability of endophytic fungi can increase the availability of nutrients to the host plant in heavy metal contaminated soil through solubilization [70]. Contamination of soil with heavy metals pollutants is mainly attributed to wastewater irrigation [41]. The result of this study also indicated that concentration of heavy metals like Ni, Cd Cu, Zn, and Pb in soil sample were higher from reference soil and World Health Organization permissible limits. The concentration of heavy metals observed in soil sample treated with waste water in the present experiment is in full agreement with previous reports [71].

Correspondingly, the inoculation of CGF-1 had a positive effect on the overall growth parameters and photosynthetic pigments (Chla, Chlb and carotenoids) in inoculated wheat plants under wastewater treatment. Likewise, in osmoregulation of wastewater treatment endophytes can play an important role by stimulating proline production, as it promotes plant growth and avoids metal stress. Proline contents in inoculated plants were higher as compared to non-inoculated plants, which is an indication to avoid stress and toxicity. Endophytic colonization also helped in improving nutrients uptake such as P and N and increased plant productivity [72, 73]. Among the minerals, the concentration of Na^+^ was higher in non-inoculated plants as compared to the CGF-1 inoculated plants under salt stress. This might be due to increased absorption of K^+^ and restriction of Na^+^ by CGF-1 inoculated plants under salt stress. The increased uptake of K^+^ leads to a high K^+^/Na^+^ ratio, which is an indication of plant resistance to high salt stress [74, 75].

Antioxidant enzymes (APX, CAT, POD and reduced glutathione GSH) can scavenge the accumulated ROS in plants to favor its defense mechanisms [76]. Oxidative stress in plants can be recognized by elevated activities of antioxidant enzymes (CAT, APX SOD and reduced glutathione GSH) under stress conditions. So, in the current study, the isolated fungal strain CGF-1 has assisted the wheat plants to produce large amounts of antioxidant enzymes under heavy metal stress. The release of these enzymes scavenged the ROS, thus enable the plant to grow normally under toxic conditions [77, 78]. Besides, the production of antioxidant enzymes, the CGF-1 colonized host plants has reduced the heavy metals uptake and consequently lowered the concentration in shoot and root tissues. The concentration of accumulated metals (Ni, Cd, Cu, Zn and Pb) in non-inoculated plants was significantly higher than those having endophytic fungal association. Similar observations concerning the low accumulation of heavy metals in endophyte associated plants have been described previously [79, 80]. Microscopic observation has revealed the presence of inoculated fungus in pericycle and cortex region of the root, which indicates the role of endophytes in metal uptake by host plants. However, further research is required to clarify the detailed mechanisms behind this phenomenon.

In conclusion, the current study concerning the use of isolated endophytic strain CGF-1 has given promising results in growth promotion of wheat plants under heavy metals stress that limited the absorption of heavy metals by host plants. Furthermore, the inoculated fungus also provided protection to the host plants from heavy metals stress by taking up the heavy metal in fungal mycelium leading to immobilization of metals. Keeping the results of the present study in view, it is highly recommended that similar approaches may be implemented to achieve sustainable agriculture on arid, saline as well as heavy metal contaminated areas.

## Supporting information

S1 Fig *S*. *surattense* plant and endophytic fungus CGF-1. Fig 1A = *S*. *surattense* plant; Fig 1B = endophytic fungus CGF-1 isolated from *S*. *surattense* plant and cultured on a Petri plate.
(TIFF)

S2 Fig Multiple sequence alignment file of 18S ribosomal DNA region. (TIF)

S3 Fig Endophytic fungal colonization potential in wheat plant tissue. (A) Wheat plants inoculated with fungal endophyte strain CGF-1 (B) Inoculated (B1-ive) and non-inoculated wheat plants (B2, B3). All plants but, control (A1+ive and B1-ive) received wastewater treatment. Plants were harvested 30 days (A1, A2 and B1, B2) or 25 days old (A3 and B3). Inoculated plants appeared stronger, green and with higher number of leaves and roots as compared to the non-inoculated plants. A1+ive: refer to strain CGF-1 inoculated 30 days old wheat plant that received no waste water treatment; while B1-ve: refers to non-inoculated 30 days old wheat plant, and it also did not receive waste water treatment. (C) Symbiotic-interaction of endophytic strain CGF-1 with shoots of wheat plants (D) Re-isolated endophytic strain CGF-1 (P. roqueforti) colonies from the shoot of lab inoculated wheat plant. (E-F) Control non-inoculated wheat plants root (E) and shoot (F) where no colonization was observed. (G) Symbiotic-interaction of endophytic strain CGF-1 with roots of wheat plants (H) Re-isolated endophytic strain CGF-1 (P. roqueforti) colonies of from roots of lab inoculated wheat plant.
(TIF)
