# Supplementary figures and images for: IAA producing fungal endophyte Penicillium roqueforti Thom., enhances stress tolerance and nutrients uptake in wheat plants grown on heavy metal contaminated soils
Source: PLoS One. 2018 Nov 29;13(11):e0208150. doi: 10.1371/journal.pone.0208150 (PMC6264496; doi:10.1371/journal.pone.0208150)

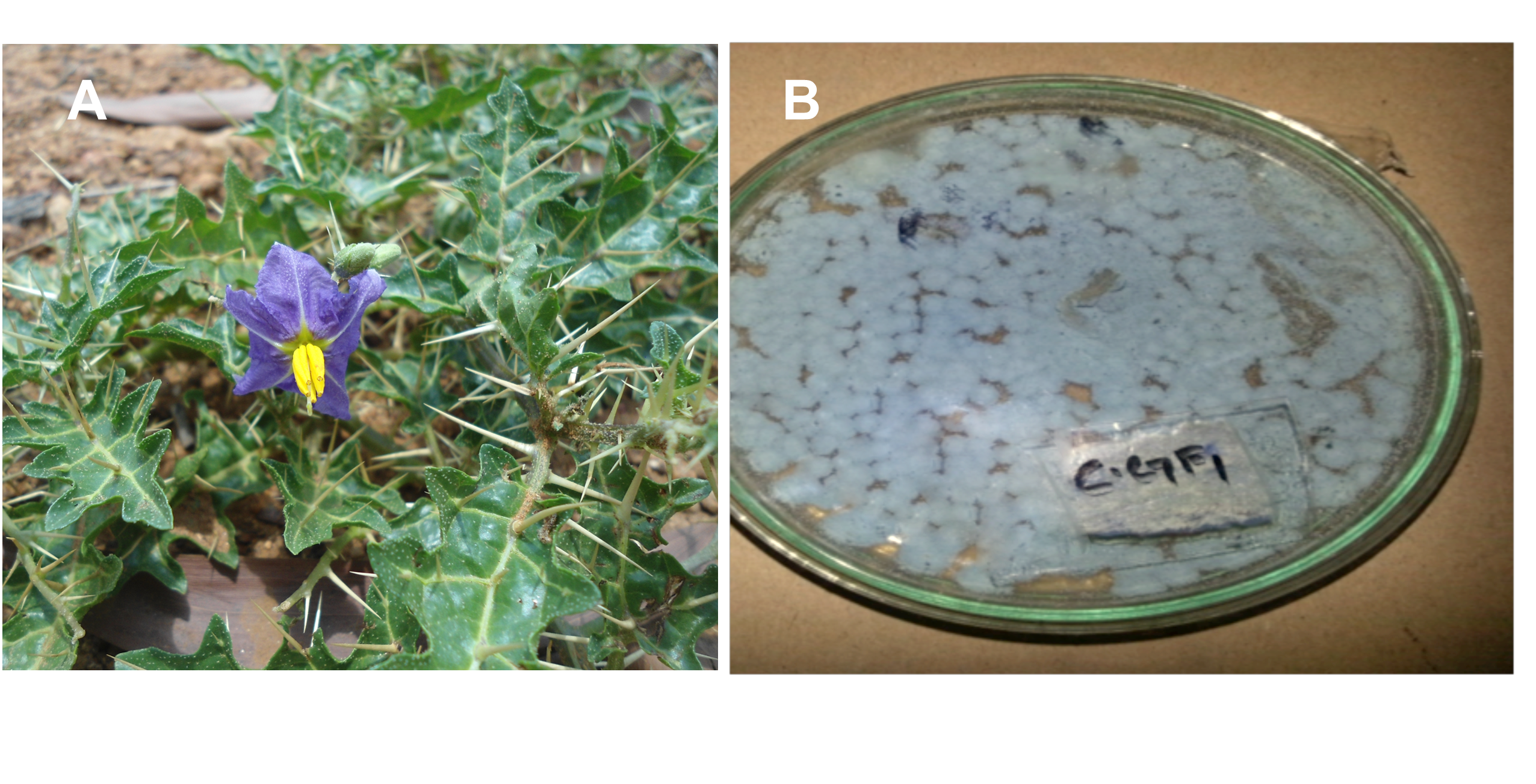

Supplement: S1 Fig — Fig 1A = S. surattense plant; Fig 1B = endophytic fungus CGF-1 isolated from S. surattense plant and cultured on a Petri plate. (TIFF) [file pone.0208150.s001.tiff]

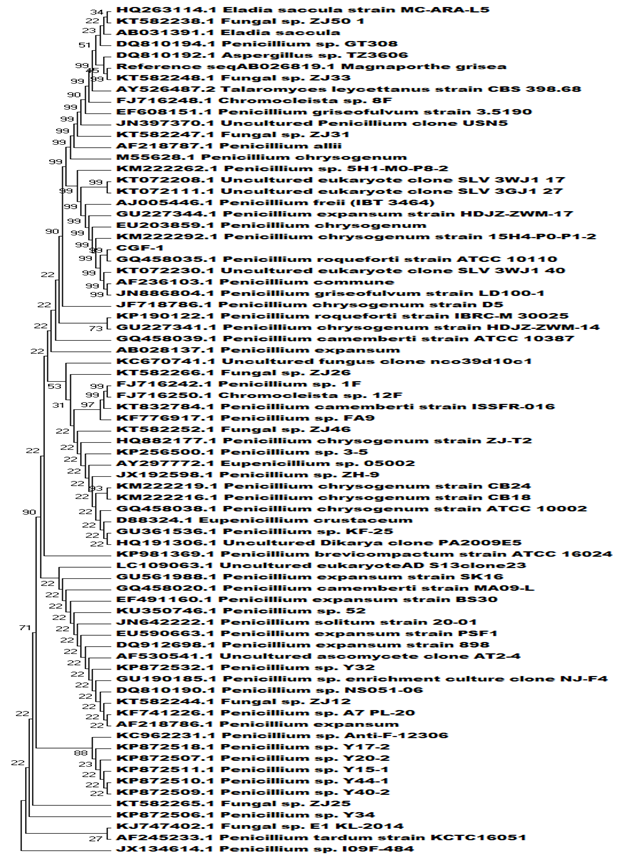

Supplement: S2 Fig — (TIF) [file pone.0208150.s002.tif]

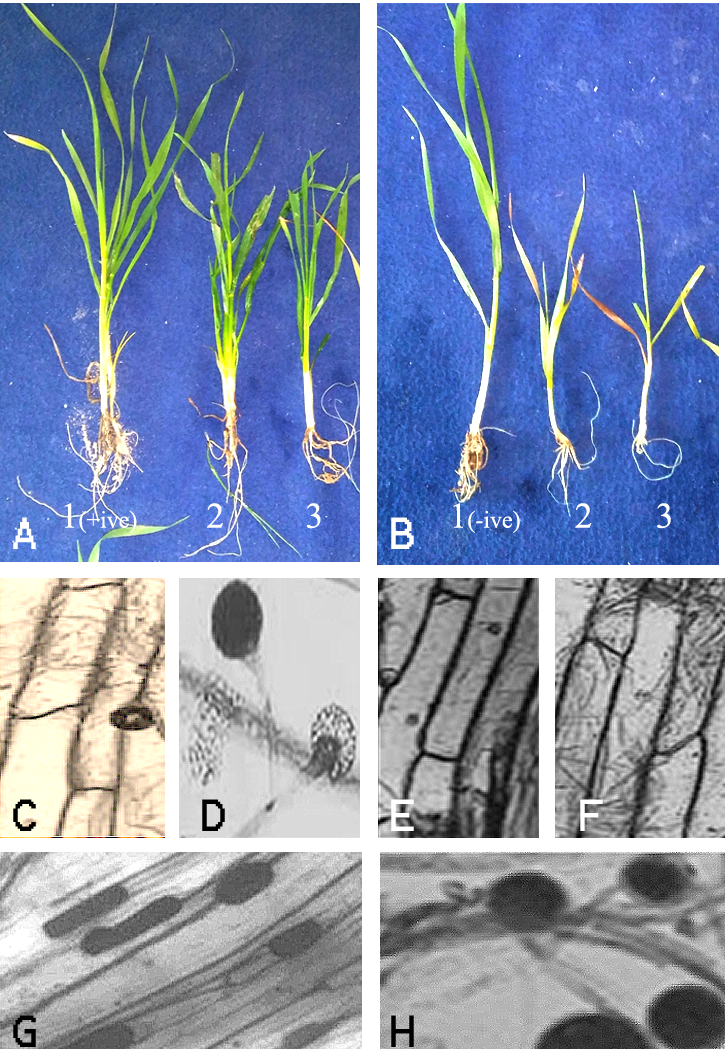

Supplement: S3 Fig — (A) Wheat plants inoculated with fungal endophyte strain CGF-1 (B) Inoculated (B1-ive) and non-inoculated wheat plants (B2, B3). All plants but, control (A1+ive and B1-ive) received wastewater treatment. Plants were harvested 30 days (A1, A2 and B1, B2) or 25 days old (A3 and B3). Inoculated plants appeared stronger, green and with higher number of leaves and roots as compared to the non-inoculated plants. A1+ive: refer to strain CGF-1 inoculated 30 days old wheat plant that received no waste water treatment; while B1-ve: refers to non-inoculated 30 days old wheat plant, and it also did not receive waste water treatment. (C) Symbiotic-interaction of endophytic strain CGF-1 with shoots of wheat plants (D) Re-isolated endophytic strain CGF-1 (P. roqueforti) colonies from the shoot of lab inoculated wheat plant. (E-F) Control non-inoculated wheat plants root (E) and shoot (F) where no colonization was observed. (G) Symbiotic-interaction of endophytic strain CGF-1 with roots of wheat plants (H) Re-isolated endophytic strain CGF-1 (P. roqueforti) colonies of from roots of lab inoculated wheat plant. (TIF) [file pone.0208150.s003.tif]
